# Research priorities in modeling the transmission risks of H7N9 bird flu

**DOI:** 10.1186/2049-9957-2-17

**Published:** 2013-08-08

**Authors:** Viroj Wiwanitkit, Benyun Shi, Shang Xia, Guo-Jing Yang, Xiao-Nong Zhou, Jiming Liu

**Affiliations:** 1 Wiwanitkit House, 10160, Bangkhae, Bangkok, Thailand; 2 Hainan Medical University, Haikou 570100, China; 3 Faculty of Medicine, University of Nis, Nis 18000, Serbia; 4 Department of Computer Science, Hong Kong Baptist University, Waterloo Road, Kowloon Tong, Hong Kong; 5 The Jockey Club School of Public Health and Primary Care, Chinese University of Hong Kong, New Territories, Hong Kong; 6 National Institute of Parasitic Diseases, Chinese Center for Disease Control and Prevention, Shanghai 200025, China; 7 Key Laboratory of Parasite and Vector Biology, MOH, Shanghai 200025, China; 8 WHO Collaborating Center for Malaria, Schistosomiasis and Filariasis, Shanghai 200025, China

**Keywords:** H7N9, Bird flu, Transmission, Modeling, Priorities

## Abstract

The epidemic of H7N9 bird flu in eastern China in early 2013 has caused much attention from researchers as well as public health workers. The issue on modeling the transmission risks is very interesting topic. In this article, this issue is debated and discussed in order to promote further researches on prediction and prevention of avian influenza viruses supported by better interdisciplinary datasets from the surveillance and response system.

## Multilingual abstracts

Please see Additional file 1 for translations of the abstract into the six official working languages of the United Nations.

## Background

The epidemic of H7N9 bird flu in eastern China in early 2013 has received much attention from researchers, as well as from public health workers. An article titled “Inferring the potential risks of H7N9 infection by spatiotemporally characterizing bird migration and poultry distribution in eastern China,” published in the journal *Infectious Diseases of Poverty* in 2013 [1], has concluded that risk mapping will provide guidance in active surveillance and control of human H7N9 infections as it would allows corresponding intervention measures to be taken. Some issues regarding the relationship between avian influenza virus (AIV) outbreaks and migration of birds has been raised by Wiwanitkit with emphasis on the prediction methodologies with various data sources, including genetic data of pathogen, immunity, and economic status of humans, as well as ecosystem factors. These issues have been addressed by Shi et al. in a systematic review on the impacts of spatiotemporal migration and contact patterns of bird species in relation to the spread of AIVs, as well as the transmission risks of AIVs to humans. This response discussion provided detailed information on how to further understand the risks of bird-to-human and human-to-human transmissions by considering various interactive impact factors, and how to better use those impact factors in the surveillance and response system with the aim of preventive actions being supported by better prediction of the transmission risks. This was further echoed by Wiwanitkit who outlines the research priorities of modeling the bird flu epidemic, which could provide guidance to the surveillance and control of AIVs. Therefore, the editor combined all parts of these discussions in one paper, in order to further promote research on the prediction and prevention of AIVs supported by better interdisciplinary datasets from the surveillance and response system. This article is the collation of these discussions.

### Viewpoint by viroj wiwanitkit: H7N9 bird flu, bird migration, and poultry distribution

Sir, the recent report by Shi et al. is very interesting [1]. In this predictive model, “bird migration” and “poultry distribution” were assessed for their interrelationship with H7N9 bird flu [1]. Using spatiotemporal risk mapping can be useful for predicting the distribution of H7N9 bird flu. Bird flu is usually the result of a worldwide outbreak due to the migration of birds. However, it is noted that “mixing of bird populations (involving residents and migratory birds) plays an important role in the patterns of disease spread [2]”.

An interesting concern is the additional factors that promote the existence of disease. Each year, birds migrate and poultry is widely distributed, but bird flu does not break out each year. The effect of migration strategies on the occurrence of the disease is also widely discussed. A report from the USA showed an interesting observation that the prevalence of the virus and its diversity did not differ in birds with different migration strategies [3]. Muzyga et al. reported that outbreaks were related to latitude, but not to migration or habitats [4]. The mismatch between timing of the outbreaks and the migration of birds can also be observed [4]. In fact, the circulation of a virus among a bird population in a specific setting is dynamic and varies on season. A recent study on the avian influenza virus (AVI) among a bird population in Ukraine is a good example which demonstrates the differences between viruses in different seasons [5]. Some previous studies mentioned the importance of immunity to infection in birds in relation to the existence of bird flu. In fact, the seasonal change of immunity is mentioned as an important pathobiology process [6]. Hegemann et al. studied how “immune function in a free-living bird varies over the annual cycle”, and found a significant variation in immunity across the annual cycle; as well as that the observation differed between years [7]. Hegemann et al. said that “fluctuating environmental conditions that vary among years [are] likely [to] contribute to the immunological variation [7]”. Hence, the occurrence of the virus seems to not be directly related to the migration of birds.

The first problem of emerging bird flu is usually the ‘unpredicted’ and ‘never predicted’ sense mutations of influenza virus that trigger cross-species infection. The existence of a virus in a new setting might be proved by a genetic study [8]. However, the detection of mutated virus is usually an observation after the outbreak of the disease [9,10]. In fact, there are some surveillance programs on the circulating virus among birds in many countries, such as Bangladesh [11], Vietnam [12], Indonesia [13], Iran [14], as well as others. Based on the data from surveillance, the isolated viruses had a broad range of genotypes indicating variability within genotypes [15]. Of interest, the mutated virus that causes emerging bird flu has never been determined despite surveillance being continuously done prior to the influenza emerging [16]. Due to unpredicted mutation, the new virus occurs and becomes the new problematic human pathogen.

Then, the humans at risk––especially those who have low immunity and live in poor communities––get attacked. This results in the wide spreading of bird flu in humans. The success in disease control at the human phase is not related to poultry control therefore, the data on ecology of birds might be limitedly useful for outbreak management in this particular phase. There are differences in the successes of disease control in different countries despite the same practice of destroying sick birds being implemented [17]. Pavade et al. reported that the countries with high-income economies had fewer and shorter bird flu outbreaks, and the eradication times for controlling the infections were also shorter [17].

In the case of an outbreak, the complexity of bio- and social- aspects of disease distribution should be kept in mind. Economic constraints have many effects on disease control [18]. In a low-income country, the local population might not be able to avoid having poultry in the backyards of their homes as it is their food source [19]. They might have close contact with poultry and its feces, and take the risk to be in contact with it, due to poverty [19]. In addition, the control of the disease cannot be successful if there is no concern for the underlying social geography. A good example is the difficulty in control bird flu in case of transboundary virus circulation among different countries with different political concepts [20]. Developing models based on both biological and socioeconomic data should be considered.

Response by Benyun Shi, Shang Xia, Guo-Jing Yang, Xiao-Nong Zhou, Jiming Liu: Transmission risks of avian influenza viruses through interactive impact factors.

The global spread of avian influenza viruses (AIVs) has always been, and continues to be, a critical threat to humans––and may result in enormous problems for public health. Professor Wiwanitkit has raised some issues concerning: (i) the relationship between the occurrence/timing of outbreaks and migration of birds, (ii) the emergence of a new virus due to unpredicted mutations, (iii) the risks humans with low immunity and low incomes face, and (iv) the impact of some biological and sociological factors to the existence of AIVs. All these issues touch the complex nature of bird flu epidemic, surveillance, and control. We would like to offer the following points for clarification, as well as for further discussion.

Generally speaking, the global spread of AIVs is a complex process which may depend on many factors, such as the location and timing of virus emergence, host species infected, viral characteristics and pathogenicity, and unforeseen stochastic events (e.g., rare cold weather) [21]. In light of the issues raised by Professor Wiwanitkit concerning the roles of the mixing of bird populations and the complexity of biological and social aspects of disease distribution, we would like to put forward the following in-depth discussion about the impacts of bird migration on the spread of AIVs, as well as the transmission risks of AIVs to humans. Specifically, we would like to emphasize three fundamental issues, namely, (i) predicting the spread of a complete (unreassorted whole genome) AIV by identifying the migration flyways of different bird species, (ii) assessing the potential emergence of new AIVs via genetic reassortment by quantifying the contact patterns of birds sharing habitats/stopovers, and (iii) understanding the risks of bird-to-human and human-to-human transmissions by considering various interactive impact factors, such as biological, demographical, socioeconomic, and environmental.

Regarding the first issue, evidence has indicated that migratory birds contribute, in part, to the spatial epidemic spread of AIVs, such as the worldwide H5N1 outbreaks [22,23]. At a global/coarse scale, the intercontinental flyways of migratory birds (e.g., the East Asian/Australia flyway), and flyways within a continent (e.g., Atlantic, Central, Mississippi, and Pacific flyways within North America [24]) have been assessed using bird banding, satellite tracking, and other techniques. To a certain extent, such pathways can help assess the introduction pathways of an AIV between continents/countries [21]. We would argue that the bird migration patterns on a finer scale (e.g., within a country/province) could be helpful for predicting the spread of a complete AIV during a short time period, like the H7N9 outbreak in eastern China from February to May 2013 [1]. Moreover, since the spread of AIVs depends on the host species that have been infected, it becomes extremely important for us to identify the flyways of individual bird species, which may exhibit heterogeneous spatiotemporal patterns. By taking into consideration both the migration of infected bird species and the possible inter-species transmissions of the virus (e.g., due to the overlapping of flyways), we will be able to quickly respond to a potential outbreak based on the location and timing of its emergence.

With respect to the second issue, migratory birds, as a natural reservoir, play important roles in the emergence of new AIVs via genetic reassortment. Reassortment can only occur when individuals are co-infected by multiple low pathogenic AIV strains. For example, recent studies have shown that the reassortment of at least four origins contributes to the emergence of the avian influenza H7N9. They are: East Asian duck origin for hemagglutinin (HA), European wild duck for neuraminidase (NA), and at least two H9N2 chicken viruses for the internal genes [25,26]. Phylogenetic analysis on the historically recorded AIVs indicates that genetic reassortment is most likely the cause of divergent gene pools of AIVs across large geographic scales [27]. Moreover, evidence has shown that mixed lineage viruses are usually found in locations where migratory flyways overlap. In this case, to assess the location and timing of the potential emergence of new AIVs, it would be helpful for us to identify migratory hotspots by quantifying the contact patterns of bird species (involving residents and migratory birds) sharing habitats and stopovers [28]. By doing so, active surveillance on the frequency of reassortment can be effectively implemented so as to prevent the outbreak of new highly pathogenic AIVs (HPAIVs) [29-31].

At present, a large quantity of observation data can be collected using various methods (e.g., bird banding and satellite tracking [5,32]) to assess the bird migration and contact patterns. For example, the United States Geological Survey (USGS) has documented more than ten thousands movements of aquatic birds between 1920 and 2013 [33]. The Cornell Lab of Ornithology has conducted a citizen science project (i.e., eBird), which has collected millions of bird observations––and the general public collated and presented the information on the location, data, species and other information throughout North America through the eBird Web application [34]. In China, similar bird observation data have been collected by the China Bird Watching Network, which is maintained by the Hong Kong Bird Watching Society [35]. All these data can help assess the migration patterns of a particular bird species and the contact patterns between different species on a finer scale. For example, machine-learning methods have been adopted to estimate the bird migration patterns from eBird observation data [36,37]. Spatiotemporal data analysis, such as exploratory models [38,39] and spatial clustering [40-42], have also been used to identify hotspots and further assess the bird contact patterns. The well-elaborated bird migration and contact patterns will in turn contribute to our better understanding of both the spread of the disease at the global level and the virus evolution at the genetic level.

Even for the aforementioned two issues considering only the prevalence of AIVs in bird species, experimental studies have shown that the susceptibility to infection varies not only by virus subtypes, but also by the species exposed [43,44].

As for the third issue concerning the bird-to-human and human-to-human transmissions, there is potential for the spread of AIVs to become more complex, and subject to a series of interactive biological, demographical, socioeconomic, and environmental factors. As commented on by Professor Wiwanitkit, unpredicted mutations may change viral characteristics and pathogenicity, as well as the mode of transmission. For example, it has been verified recently that one strain of the H7N9 virus appears to spread easily through the air between ferrets due to mutations, which spotlights the risks of mammal-to-mammal spread [45]. Meanwhile, age-specific and location-specific human immunities to AIVs, which may be highly related to historical exposures of the host population, are also of critical importance for the spread of the disease [46-49]. Once the human-to-human transmission is confirmed, more impact factors should be carefully considered, such as human mobility and contact patterns [50,51], and social influence [52-54]. We agree with Professor Wiwanitkit in this regard and believe that it would be essential for us to study the spread of AIVs from a novel, complex systems perspective.

In summary, quantifying bird migration and contact patterns on a finer scale will contribute to both the prediction of the spread of the disease at the global level and the assessment of potential reassortment of AIVs at the genetic level. Accordingly, the results will offer public health managers new insights into (i) how to implement active surveillance before the outbreak of an AIV, and (ii) how to quickly respond to the outbreak of an AIV at the beginning of its spread. To further carry out systematic modeling of the global spread of bird flu and hence understand the impacts of interactive factors across different scales, interdisciplinary research efforts and international collaboration will be extremely important.

### Response by viroj wiwanitkit: research priorities of modeling transmission risks of bird flu

Prof Liu et al. gave very useful information and discussions on the “transmission risks of avian influenza viruses through interactive impact factors.” In conclusion, Prof Liu et al. mentioned the usefulness of “quantifying bird migration and contact patterns on a finer scale” in two main ways: (a) an alternative way for active surveillance of the possible outbreak of AIVs, and as (b) a tool or a guidance for the early-phase predictions of an outbreak. Without a doubt, the best model has to be further developed. A model which includes the impacts of interactive factors across different scales is our aim.

As Prof Liu et al. noted, interdisciplinary work and international collaboration is needed for the success in developing such a model that can be useful worldwide. In fact, this is the concern that has been raised by some experts [55-57]. For example, the group of El-Zoghby et al. in Egypt called for “global collaboration” to set an effective surveillance system to fight AIVs [55]. Abdelwhab et al. also interestingly mentioned that there could be no success in controlling the situation of an AIV outbreak in a nation, which was in this case Egypt, without international collaboration [56]. In fact, the attempt to form the collaboration has already been made. A good example is the “The 2008 FAO-OIE-WHO Joint Technical Consultation on Avian Influenza at the Human Animal Interface.” [58,59].

There is no doubt that there is still a long way to go, and many further efforts and challenges ahead for medical scientists and analytics researchers. It is necessary to plan for success to be achieved. At present, the research priorities for the current situation should focus on the following:

A. The collection of data from each setting is very important. Since the model has to be based on data, more data mean a more accurate model. To get the best data in each setting, interdisciplinary teams should work to collect the data in a holistic way, covering biological, physical, social, and humanistic facets [60]. A good example is the work published by Vanthemsche et al. [61].

B. Bridging or fusing data from different sources to form the final “homogenized” data has to be done. This is the first step to developing a perfect, systematic model of the global spread of bird flu. A good example is the attempt by Breton et al. to use an in silico technique for manipulating international grid data for the integration of existing data sources towards a global surveillance network [62].

C. An effective predictive model has to be developed. The important key for success of the derived model for prediction of the situation is its sensitivity to correspond to the dynamic nature of an AIV outbreak. The use of advanced mathematical modeling and innovative informatics techniques might be a way to predict “risk”, not “existence”, for the occurrence of an outbreak early. To correspond to a new emerging influenza, the model that can perform an immediate identification of an impending epidemic and pandemic strains can be hugely successful in combating influenza [63].

Finally, it should be emphasized that the model, and the surveillance and response system, should be continuously operated and improved. The sustainability is very important.

### List of research priorities

• Data Collection: A rich set of relevant data across different scales (e.g., biological, demographical, socioeconomic, and environmental data) should be continuously collected, carefully maintained, and publicly shared through an interdisciplinary team effort.

• Data Processing: Advanced data analytics technologies (e.g., machine learning, data mining, and spatiotemporal exploratory methods) should be developed to process the available large-scale, sparse, or even biased datasets.

• Complex Systems Modeling: A systematic modeling approach, which bridges data from various sources at different scales, should be developed to best understand the transmission risks of AIVs to humans from a complex systems’ perspective.

• Risk Prediction: Prediction models with both short-term and long-term estimations for bird-to-human and human-to-human transmission risks should be developed and deployed so as to offer public health authorities and practitioners with new insights into, as well as new tools for, both active surveillance and infection control of AIVs.

• Simulation and Optimization: Advanced computer simulation tools and optimization approaches should be developed and deployed to (i) envision the real-world situations for the potential spread of AIVs, and (ii) provide optimal decision-making strategies for public health authorities and practitioners to quickly respond to the emergence of AIV outbreaks. All above-mentioned systems should be continuously operated and improved based on the data from dynamically changing environments.

## Competing interests

The authors declare that they have no competing interests.

## Authors’ contributions

VW draft the Part 2 and Part 4, BS and JL draft the Part 3 and Box 1. XNZ draft the Part 1. VW, BS, SX, GJY, XNZ, JL improved the whole manuscript and approved the final version.

## Supplementary Material

Additional file 1 Multilingual abstracts in the six official working languages of the United Nations. Click here for file
