# Supplementary material for: Research priorities in modeling the transmission risks of H7N9 bird flu
Source: Infect Dis Poverty. 2013 Aug 8;2:17. doi: 10.1186/2049-9957-2-17 (PMC3751567; doi:10.1186/2049-9957-2-17)
Supplement: Additional file 1 — Multilingual abstracts in the six official working languages of the United Nations. [file 2049-9957-2-17-S1.pdf]

Translation of the abstract into the six official working languages of the United Nations

أولويات البحث في وضع نماذج مخاطر انتقال العدوى لفيروس أنفلونزا الطيور **H7N9**

Viroj Wiwanitkit, Benyun Shi, Shang Xia, Guo-Jing Yang, Xiao-Nong Zhou, Jiming Liu

تلخيص

لفت الوباء المتوطن لفيروس H7N9 لإنفلونزا الطيور في شرق الصين عام 2013 انتباه كثير من الباحثين والعاملين بالصحة العامة. ويعد موضوع وضع نماذج مخاطر الانتقال والعدوي موضوع شيق للغاية. وسوف نقوم في هذه المقالة ببحث هذا الموضوع ومناقشته من أجل تطوير مزيد من الأبحاث الخاصة بالتنبؤ والوقاية من فيروسات أنفلونزا الطيور مدعومة بقواعد بيانات نظامية أكثر جودة من نظم المراقبة والاستجابة.

Translated from English version into Arabic by Mohamed Gaafar, through

TRANSLATORS
WITHOUT BORDERS

H7N9 禽流感病毒传播风险建模的优先研究领域

Viroj Wiwanitkit, Benyun Shi, Shang Xia, Guo-Jing Yang, Xiao-Nong Zhou, Jiming Liu

摘要

2013 年初中国东部 H7N9 禽流感疫情引起研究人员和公共卫生工作者的极大关注。传播风险建模成为重点关注内容。本文对这一问题进行了辩论和讨论，旨在进一步完善监测应对系统的跨学科数据集，以支持禽流感病毒预警和预防措施研究的深入开展。

Translated from English version into Chinese by Yang Pin, through

CHINESE CENTER FOR DISEASE CONTROL AND PREVENTION
NATIONAL INSTITUTE FOR PARASITIC DISEASES

Les priorités de recherche en modélisation des risques de transmission de la grippe aviaire H7N9

Viroj Wiwanitkit, Benyun Shi, Shang Xia, Guo-Jing Yang, Xiao-Nong Zhou, Jiming Liu

Résumé

L'épidémie de grippe aviaire H7N9 en Chine orientale au début de l'année 2013 a attiré l'attention des chercheurs ainsi que les travailleurs de la santé publique. La question sur la modélisation des risques de transmission est un sujet très intéressant. Dans cet article, cette question est débattue et discutée afin de promouvoir de nouvelles recherches sur la prévision des virus de la grippe aviaire soutenues par de meilleurs ensembles de données inter multidisciplinaires du système de surveillance et de réponse.

Translated from English version into French by Johanne Jean-Maître, through

TRANSLATORS
WITHOUT BORDERS

Научно-исследовательские приоритеты в моделировании рисков передачи вируса птичьего гриппа H7N9

Вирож Виваниткит, Бениун Ши, Шан Ксиа, Гуо-Джин Янг, Ксиао-Нонг Джоу, Джиминг Лиу

Краткий обзор

Эпидемия птичьего гриппа H7N9 в Восточном Китае в начале 2013 года привлекла внимание исследователей и работников системы здравоохранения. Проблема моделирования рисков передачи инфекции сама по себе очень интересна. Обсуждение вопроса в данной статье позволит стимулировать дальнейшие исследования в области предсказания и предотвращения распространения вирусов птичьего гриппа за счет расширенных межотраслевых баз данных, пополненных благодаря использованию систем наблюдения и реагирования.

Translated from English version into Russian by Irina Zayonchkovskaya, through

TRANSLATORS WITHOUT BORDERS

Prioridades de investigación en ejemplos de riesgos de transmisión de la gripe aviar H7N9

Viroj Wiwanitkit, Benyun Shi, Shang Xia, Guo-Jing Yang, Xiao-Nong Zhou, Jiming Liu

Resumen

La epidemia de gripe aviar H7N9 en el este de China a principios de 2013 ha captado la atención de muchos investigadores, así como de trabajadores de la sanidad pública. La idea de ejemplificar los riesgos de transmisión es un tema muy interesante. En este artículo se debate y se analiza el tema con el fin de promover nuevas investigaciones sobre la predicción y la prevención de virus aviares de la gripe, apoyadas por mejores datos interdisciplinares derivados del sistema de vigilancia y respuesta.

Translated from English version into Spanish by helena2501, through
